# Congenital Peritoneal Encapsulation Limiting Generalized Peritonitis: A Case Report of a Human Kangaroo Sac

**DOI:** 10.7759/cureus.113664

**Published:** 2026-07-30

**Authors:** Richa Jha, Riya

**Affiliations:** 1 General Surgery, GS Medical College and Hospital, Hapur, IND

**Keywords:** congenital anomaly, emergency laparotomy, gastric perforation, localized peritonitis, peritoneal encapsulation

## Abstract

Congenital peritoneal encapsulation (CPE) is a very rare entity that occurs due to atypical embryonic development of the gastrointestinal tract, resulting in an extraperitoneal membrane that partially or completely encloses the small intestine. It is an exceptionally rare condition that is usually asymptomatic and is identified incidentally during surgery or autopsy. We report a case of a 34-year-old male patient who presented with stable vitals and features of localized peritonitis of the left upper abdomen. The chest X-ray (posteroanterior (PA) view) and abdominal X-ray (erect view) showed gas under the diaphragm, and contrast-enhanced computed tomography (CECT) of the abdomen was suggestive of pneumoperitoneum. Exploratory laparotomy confirmed the diagnosis of CPE with gastric perforation. The rare presence of peritoneal encapsulation may have limited the spread of peritoneal contamination, making the patient present with only features of localized peritonitis and less severe symptoms.

## Introduction

Congenital peritoneal encapsulation (CPE) is a very rare developmental anomaly that occurs around the 12th week of fetal development, wherein an additional layer of peritoneum forms around the small bowel, which partially or completely surrounds it [1,2,3]. This accessory membrane anchors the ascending and descending colon sideways, the transverse mesocolon, and the posterior parietal peritoneum above and below, respectively [4]. Clinically, it is often discovered accidentally during surgery or in cases of bowel obstruction. Asymptomatic cases may remain undiagnosed and are only discovered during autopsy. Less than 50 cases of CPE have been reported in medical documentation [1]. There is a 5:3 male preponderance for CPE, of which only 14 cases have caused acute bowel obstruction [5].

Herein, we report an exceptionally rare case of localized peritonitis resulting from gastric perforation associated with CPE, which was managed in our tertiary teaching hospital.

## Case presentation

**Figure 1:**
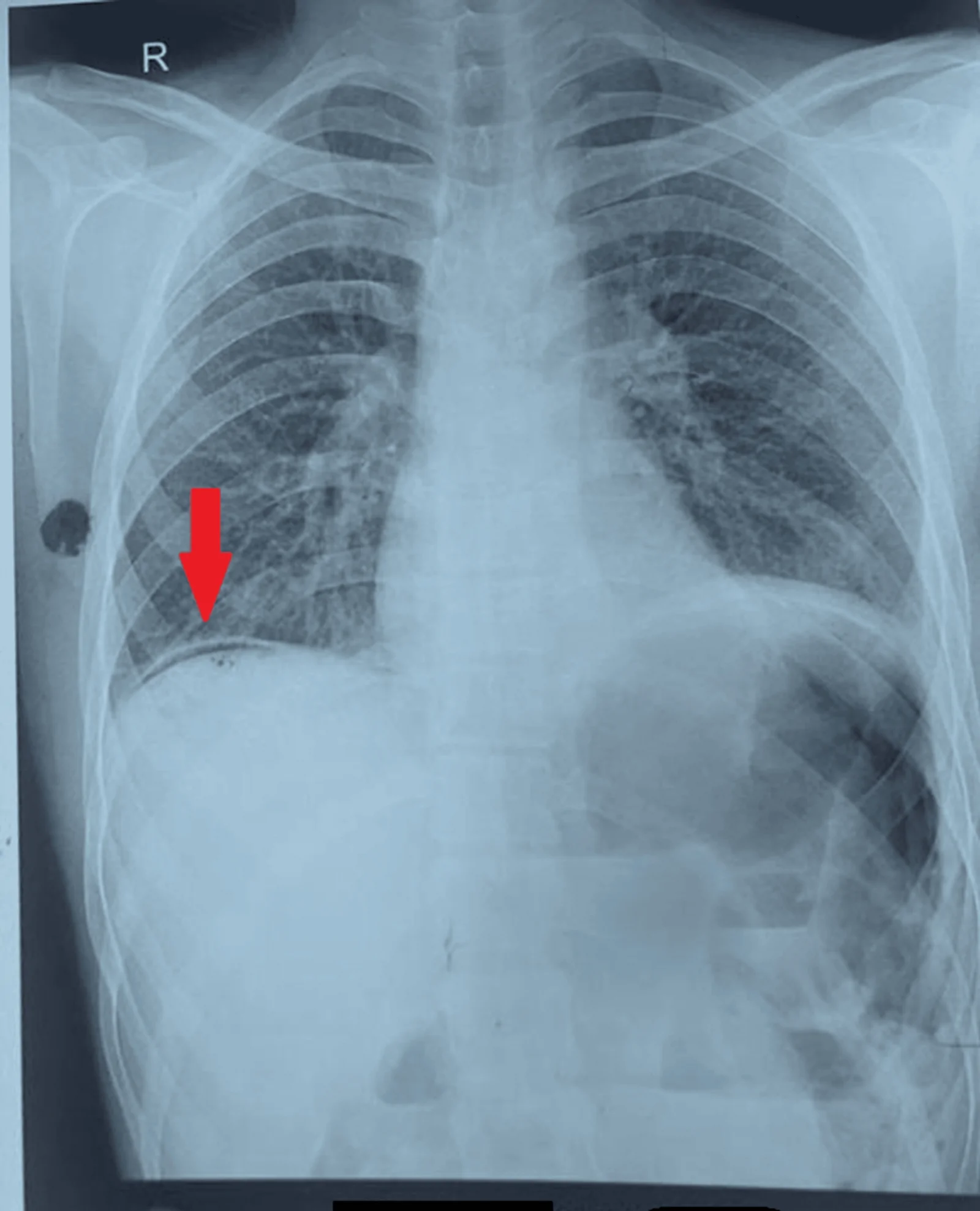
Chest X-ray (posteroanterior (PA) view) showing gas under the diaphragm (red arrow).

**Figure 2:**
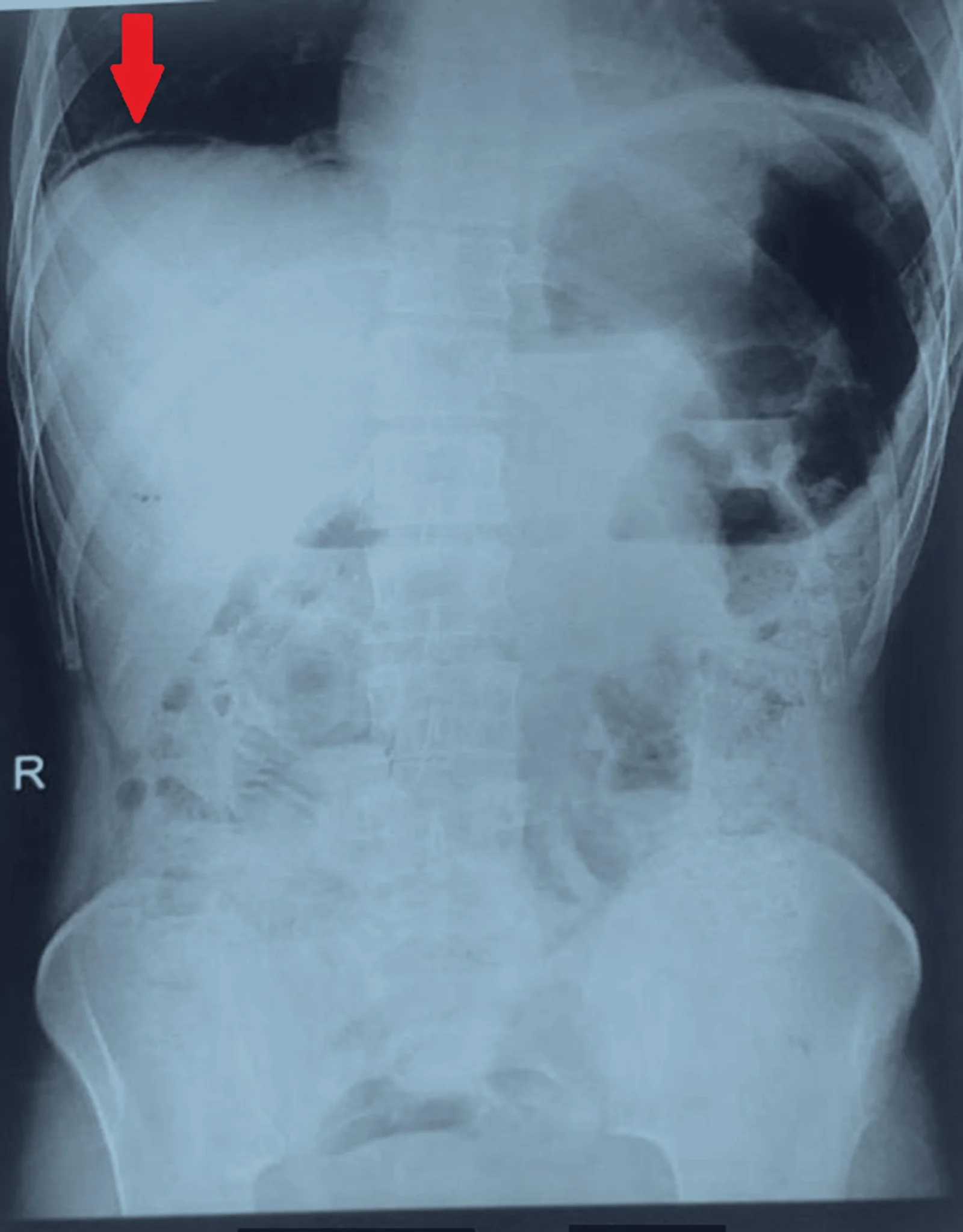
Erect abdominal X-ray showing gas under the diaphragm, suggestive of pneumoperitoneum (red arrow).

**Figure 3:**
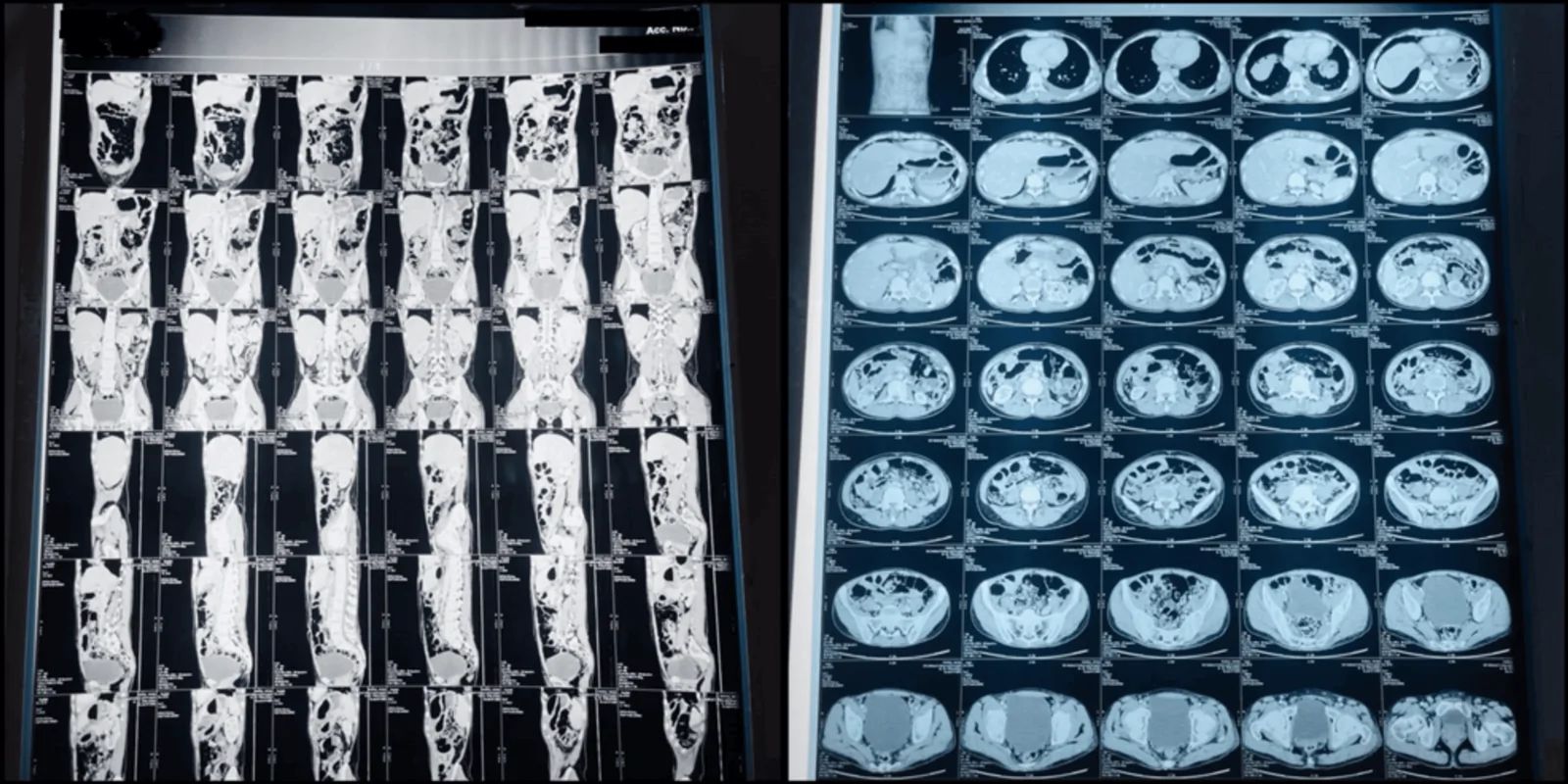
Contrast-enhanced computed tomography (CECT) of the whole abdomen showing multiple air foci in the peritoneal cavity (pneumoperitoneum), predominantly in the upper abdomen, with minimal to mild free fluid in the peritoneal cavity and minimal pleural effusion in the left thoracic cavity.

A 34-year-old male patient presented to our emergency department with the chief complaints of abdominal pain for four days, which was associated with one episode of vomiting and fever. The patient had not passed flatus or stool for two days. The patient had no history of comorbidities. The patient had no past surgical history, medication history, or any significant family history. No history of any addiction was also noted. On examination, the patient was vitally stable. On abdominal examination, the patient had localized guarding and rigidity of the left upper quadrant of the abdomen. Chest X-ray (posteroanterior (PA) view) (Figure 1) and erect abdominal X-ray (Figure 2) showed gas under the diaphragm, which was suggestive of pneumoperitoneum. Contrast-enhanced computed tomography (CECT) of the whole abdomen was suggestive of multiple air foci in the peritoneal cavity (pneumoperitoneum), predominantly in the upper abdomen. Minimal to mild free fluid was observed in the peritoneal cavity, along with minimal pleural effusion in the left thoracic cavity (Figure 3).

**Figure 4:**
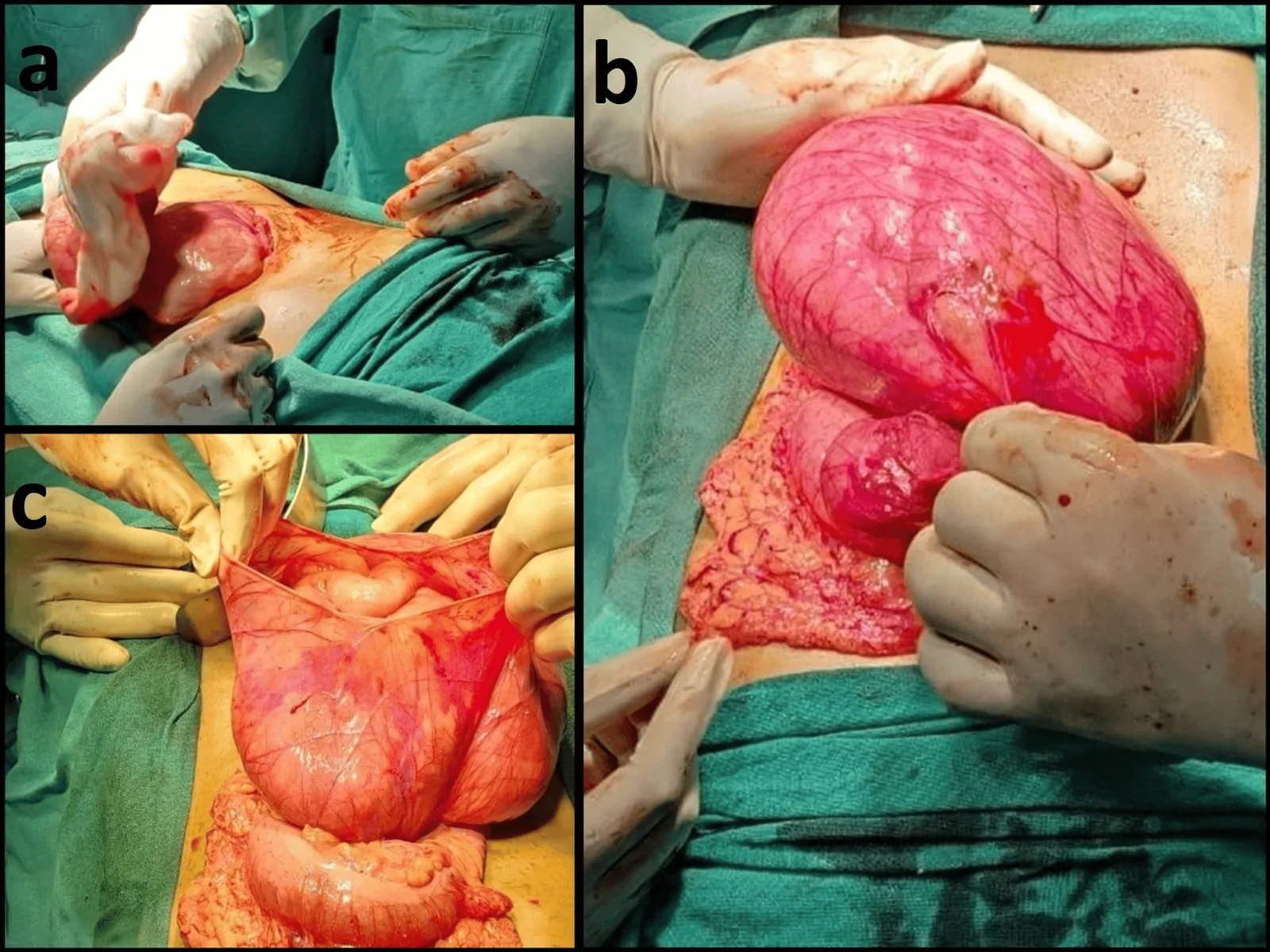
Visualization of the congenital peritoneal encapsulating sac. (a) Initial identification of the congenital peritoneal encapsulating sac. (b) Complete externalization of the congenital peritoneal encapsulating sac. (c) Opening of the peritoneal encapsulating sac and identification of the entire small bowel inside.

**Figure 5:**
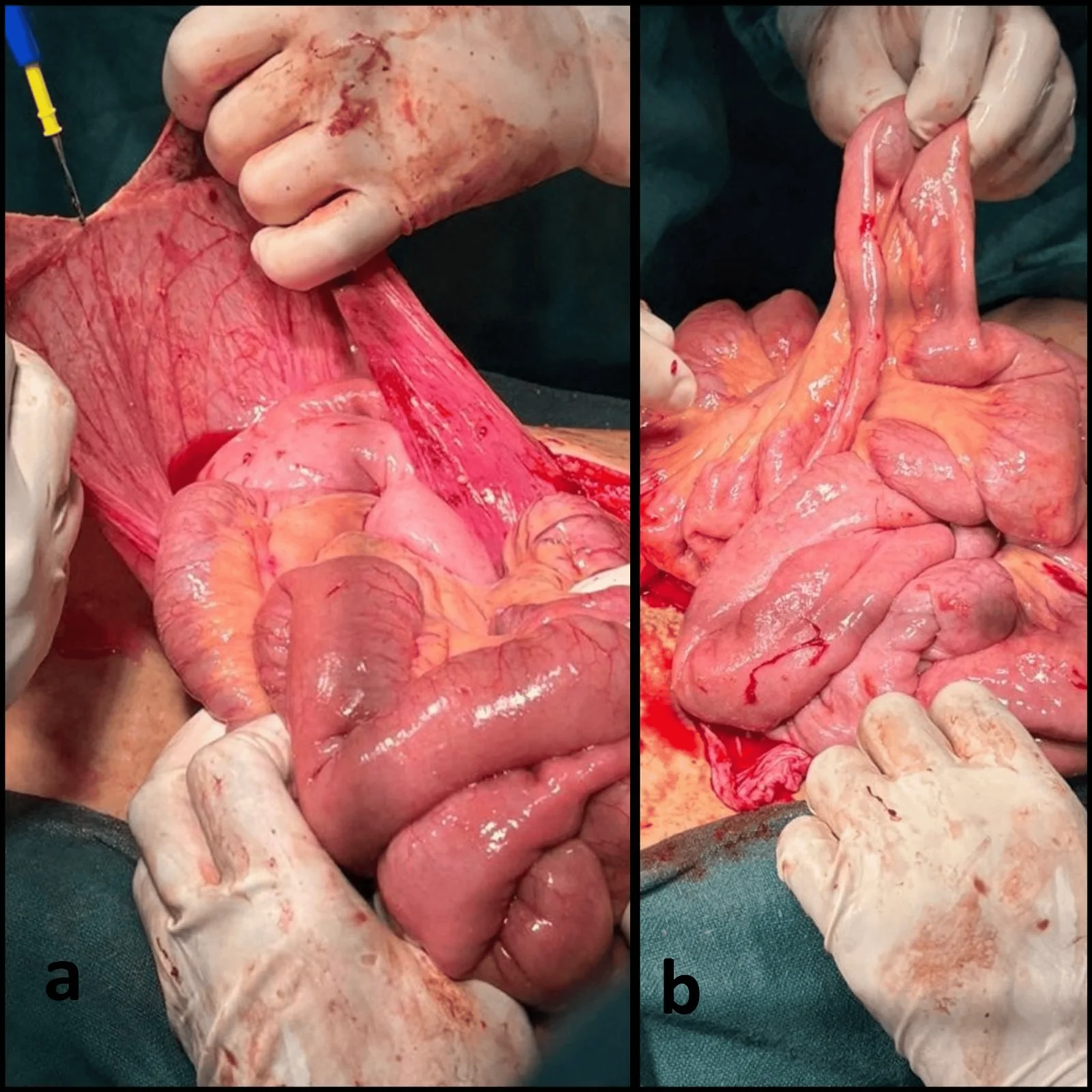
Inspection of the peritoneal sac for its contents. (a) Extending the opening of the peritoneal sac and releasing the small bowel from inside. (b) Inspecting the entire bowel for any perforation.

**Figure 6:**
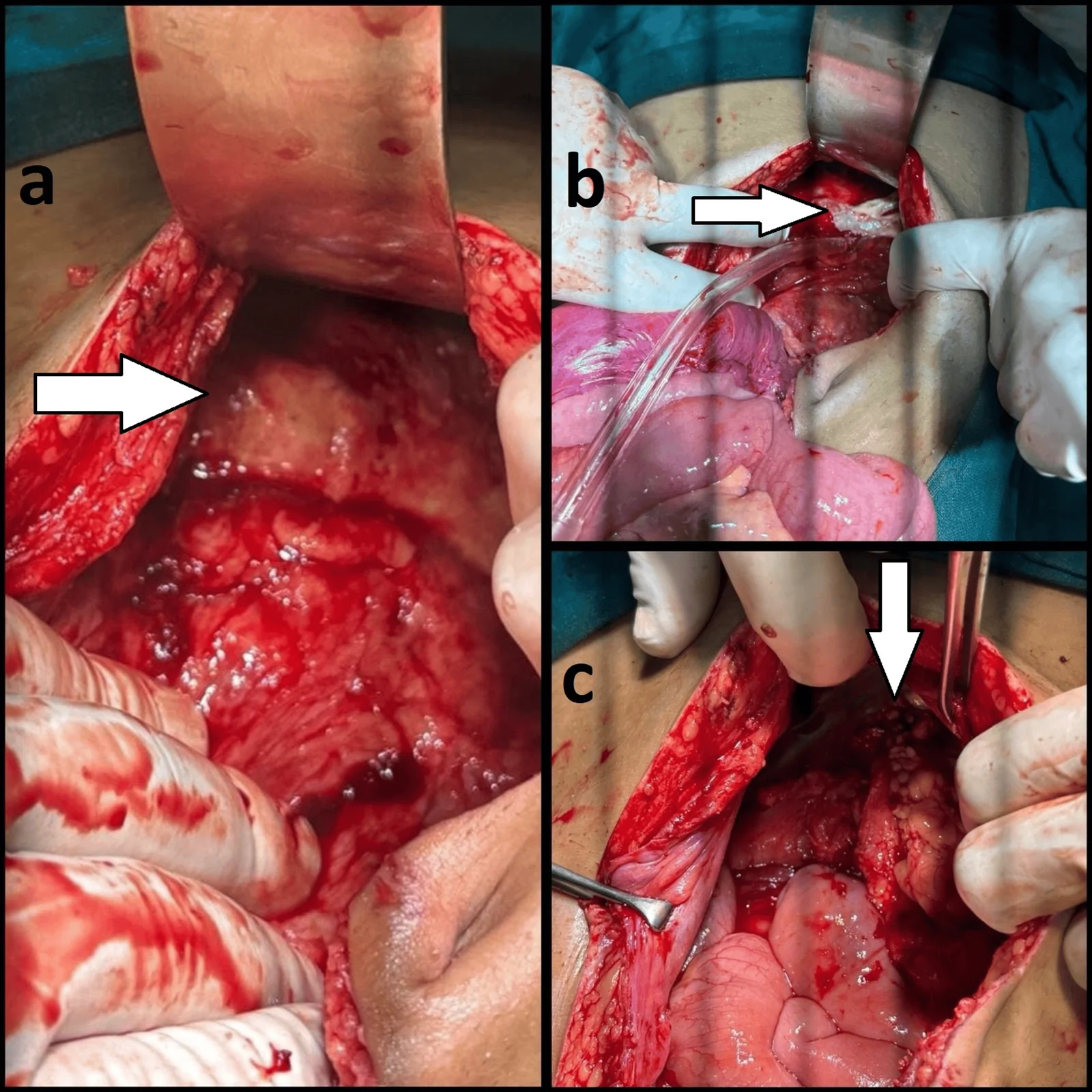
Identification of the gastric perforation and surgical repair. (a) Identifying the gastric perforation (white arrow). (b) Suctioning of pus and slough (white arrow) around the perforation site. (c) Modified Graham’s omental patch repair performed for gastric perforation (white arrow).

**Figure 7:**
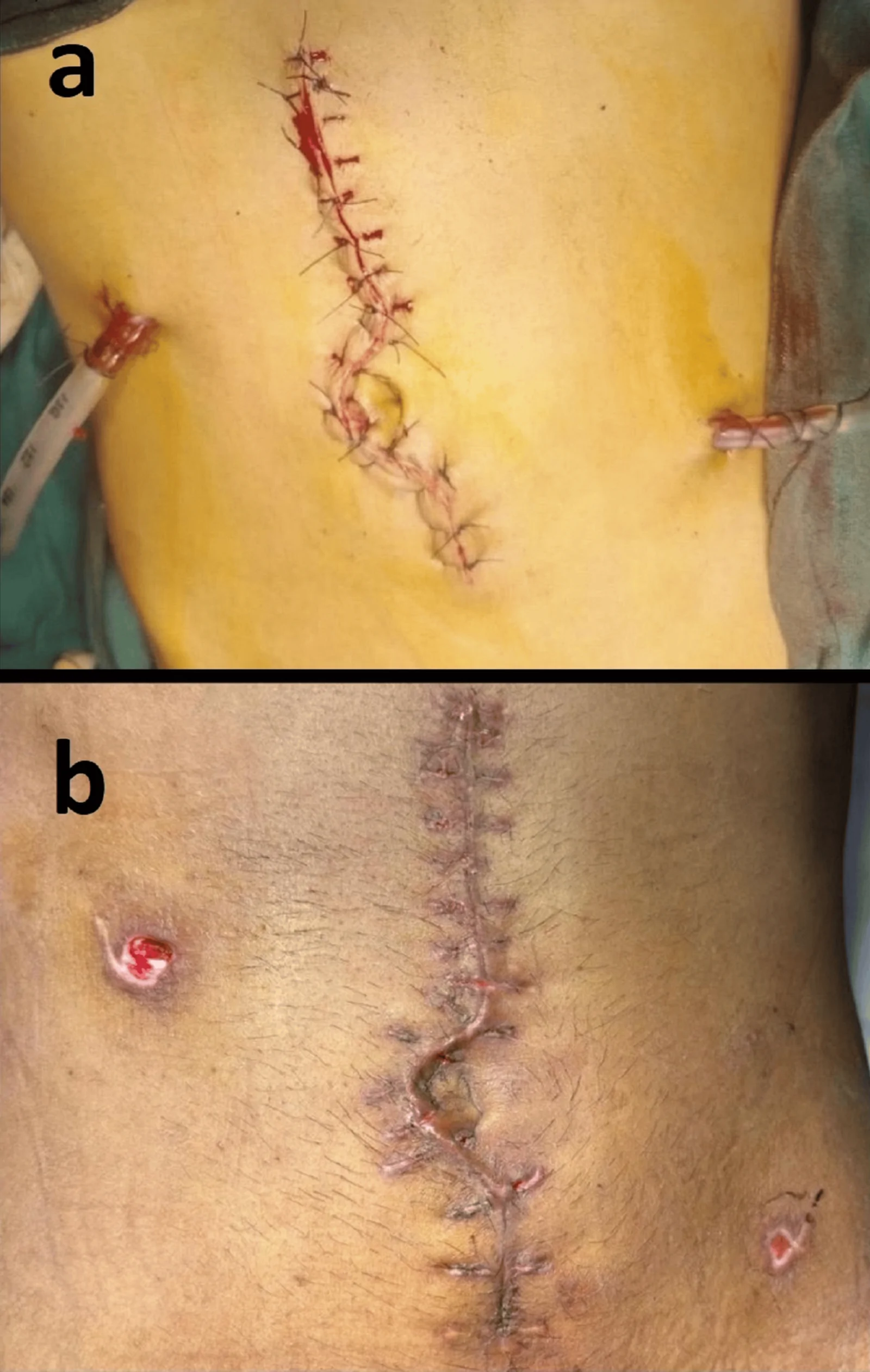
Surgical incision and postoperative scar. (a) Skin closure with bilateral drains in situ after the surgical procedure. (b) Postoperative day 10 after suture and drain removal.

Emergency exploratory laparotomy was performed after obtaining written informed high-risk consent. An anterior prepyloric perforation of the stomach, approximately 1 × 1 cm in size, was seen with pus flakes present. The peritoneal contamination was limited to localized purulent fluid around the perforation. Pus was collected and sent for culture and sensitivity. A thin membranous sac was visualized enclosing only the small intestine, which was normal in contour (CPE). The rest of the bowel was within normal limits (Figures 4-7).

The peritoneal sac that was identified was attached laterally to the ascending and descending colon, above to the transverse colon, and below to the pelvic peritoneum. The peritoneal sac was opened, and the small bowel was released into the peritoneal cavity. The gastric perforation was repaired using the modified Graham’s omental patch repair. The postoperative period was uneventful. Klebsiella pneumoniae species was isolated in the pus culture and sensitivity report, and the patient was started on antibiotics accordingly. The patient was discharged with stable vitals and a healthy incision line. Follow-up after one week and after one month was uneventful, and the patient did not have signs of any postoperative complications. Written informed consent was also obtained for the publication of the clinical photographs, and there was no conflict of interest. 

## Discussion

Bowel encapsulation is fundamentally classified into two categories: congenital and acquired. In 1868, Cleland was the first to report congenital peritoneal encapsulation [6]. This uncommon developmental anomaly arises during the 12th week of gestation due to abnormal midgut migration back into the abdominal cavity. This developmental anomaly causes persistence of the umbilical vesicle covering over the small intestine despite normal positioning at the umbilical pedicle, resulting in the formation of an accessory peritoneal membrane that partially or completely encases the small intestine within an additional sac [4].

Acquired peritoneal encapsulation encompasses two main subtypes: *Idiopathic Abdominal Cocoon* (AC) was originally reported by Foo et al. in 1978 [7]. This condition predominantly affects young adolescent females in tropical and subtropical regions. The underlying pathophysiology involves retrograde menstruation accompanied by secondary viral infection, retrograde peritonitis, and other gynecological infections, which lead to cell-mediated tissue damage [1,7]. Encapsulation of the small bowel by a thick fibrocollagenous membrane forms a cocoon-like sac with internal adhesions. *Encapsulating peritoneal sclerosis* (EPS) was first stated in 1907 by Owtschinnikow [8]. Here, the fibrotic membrane is secondary to various inflammatory abdominal conditions. Peritoneal dialysis is the most common etiological factor, followed by peritoneal shunts, malignancy, tuberculosis, and systemic diseases, including systemic lupus erythematosus, sarcoidosis, and Mediterranean fever [1,8].

Peritoneal encapsulation is typically discovered incidentally during laparotomy or at autopsy. In our case, CPE was identified during exploratory laparotomy, where it paradoxically benefited the patient by limiting the spread of peritoneal contamination, resulting in localized peritonitis. This containment preserved the patient's hemodynamic stability and prevented the progression of shock and generalized peritonitis.

## Conclusions

CPE is an extremely rare congenital anomaly characterized by the partial or total enclosure of the small bowel within the peritoneal membrane. It varies from abdominal cocoon and EPS, as these conditions arise due to secondary inflammatory processes. Awareness of this condition is crucial, as it frequently goes unrecognized, inadequately managed, and inappropriately treated.

Only a limited number of cases regarding CPE have been documented in the literature, particularly those involving localized peritonitis secondary to gastric perforation.

Given that peritoneal encapsulation structurally mimics a kangaroo's pouch, we have titled our case report *Human Kangaroo Sac*.
